# A Study in Wad Madani, Sudan: Are We Documenting Operation Notes Effectively?

**DOI:** 10.7759/cureus.66544

**Published:** 2024-08-09

**Authors:** Ahmed Mohamed, MohammedElhassan Abdalla

**Affiliations:** 1 Department of Orthopaedics, Gezira Centre for Orthopedic Surgery and Traumatology, Wad Madani, SDN

**Keywords:** training programs, standards, royal college of surgeons, operation notes, documentation

## Abstract

Background: Operative notes represent the critical record of a surgical procedure, encompassing comprehensive details encountered throughout the operation. Recognizing the importance of comprehensive documentation, the Royal College of Surgeons (RCS) developed the Good Surgical Practice guidelines, which emphasize accurately recording every procedure and specifying the necessary parameters for each operative note. These guidelines help maintain high standards of surgical care and patient safety.

Methods: A retrospective review of 88 orthopaedic surgery operative notes for fracture neck of femurs was conducted at Gezira Centre for Orthopedic Surgery and Traumatology (GCOST) from March 12 to May 28, 2022. The review assessed 18 parameters against RCS guidelines. Statistical analysis was performed using Statistical Product and Service Solutions (SPSS, version 25.0; IBM SPSS Statistics for Windows, Armonk, NY), which facilitated comprehensive data examination.

Results: In 37 cases (42.05%), the operation notes were written by a medical officer. In 29 cases (32.95%), an orthopaedic resident authored the notes. A specialist documented the notes in 21 cases (23.86%), and a consultant wrote the notes in one case (1.14%). Over 90% of the notes included surgeon and assistant names, procedure names, operative diagnoses, operative procedures, prosthesis details, deep vein thrombosis (DVT) and antibiotic prophylaxis, and signatures. The name of the theatre anaesthetist, elective/emergency details, and additional procedures with reasons were absent in all notes. Less than 50% of the notes documented the time of the procedure, type of incision, operative findings, anticipated blood loss, closure technique specifics, and complications.

Conclusion: The study emphasizes the shortcomings in the operating notes, underscoring the necessity for training initiatives to enhance the recording by medical officers and orthopaedic trainees. Implementing structured templates that adhere to RCS standards can improve the comprehensiveness and consistency of operating notes, effectively resolving existing discrepancies. Regular audits and feedback sessions are essential for identifying and rectifying persistent issues. It is recommended to arrange workshops and seminars to educate medical officials and trainees on the skills of efficient note-taking and thorough documentation procedures.

## Introduction

An operation note serves as a medicolegal record of a patient's care and is crucial for maintaining continuity of care between the operating team and other colleagues [1]. Effective medical record-keeping is a pivotal component of patient management. It helps in the scientific evaluation of the patient profiles, aiding in the analysis and formulation of treatment plans. Surgeons emphasize the critical importance of maintaining optimal note quality for precise surgical patient management, as they rely on these comprehensive records to make critical decisions tailored to individual cases. The National Confidential Enquiry into Patient Outcome and Death (NCEPOD) [2] has provided evidence that poor documentation can have a detrimental impact on postoperative care at various stages, including immediately postoperatively, predischarge, and subsequent patient care.

Legibility issues arise because most operative notes, in many parts of the world, are handwritten [3]. The switch to typed notes has shown promise in improving documentation quality and reducing errors. Additionally, computerized notes offer numerous benefits over handwritten ones, such as enhanced readability, more effective time management, and smoother departmental communication within healthcare facilities. However, this shift requires a significant investment of resources, including personnel training and computer infrastructure installation.

An operating team member is required to complete notes as soon as possible following an operation [4]. According to the most recent recommendations from the Royal College of Surgeons of England (RCS), notes can be written by hand or, better yet, typed [4]. Additionally, the notes should go with the patient to the ward and recovery area and should provide enough information to allow for continued care by a different physician.

The RCS developed Domain 1.3 of the Good Surgical Practice guidelines, which highlights the importance of accurately documenting every procedure carried out and specifies the parameters that each operative procedure must have [4]. Along with a record of the serial numbers of the prosthesis that were implanted, it is crucial to make sure that any intraoperative photos are appended to the note or saved to a picture archiving and communication system (PACS).

The purpose of this study was to evaluate the quality of operative notes at our centre against the recognized standard, identify pitfalls, and make recommendations to improve operative notes.

## Materials and methods

This retrospective study aimed to evaluate the quality of operative notes in accordance with the guidelines set by the RCS. The research was conducted at the Gezira Centre for Orthopedic Surgery and Traumatology (GCOST), located in Wad Madani, Sudan. The study period extended from March 12 to May 28, 2022, providing a comprehensive analysis of the documentation practices over this time frame. Its main objective was to determine whether the operative notes adhered to the RCS's standards, which prioritize accuracy, comprehensiveness, and clarity. The efficiency of surgical teams and the quality of patient care can be significantly enhanced by adhering to these guidelines.

The researchers concluded that ethical clearance was unnecessary due to the retrospective nature of this study, which involved the analysis of previously recorded operative notes rather than the collection of new data or the implementation of interventions. The decision was made on the basis that the study did not involve any new treatments or procedures nor did it involve direct interaction with patients.

The study specifically targeted operative notes for patients who underwent surgical interventions related to fractures of the neck or femur within the study timeframe. Both male and female patients were considered eligible for inclusion. However, since the Gezira Centre also performs plastic and neurosurgical procedures, these were excluded from the study to maintain focus on the relevant orthopaedic cases. Additionally, any procedures not related to the fixation of fractured femoral necks were not included. This exclusion criterion extended to surgeries addressing other conditions. Patients who had experienced multiple traumas affecting other body parts and those who had undergone revision surgeries for previously treated femoral neck fractures were also excluded.

During the two-and-a-half-month study period, a total of 88 operative notes recorded by the surgical team were reviewed. All statistical analyses were conducted using Statistical Product and Service Solutions (SPSS, version 25.0; IBM SPSS Statistics for Windows, Armonk, NY). A descriptive statistical analysis was conducted to interpret the results. Data collection adhered to the RCS operation note writing guidelines, with all information directly transcribed into a structured format comprising two fundamental checking components: 'Yes' and 'No'.

## Results

From the 88 operative notes for neck fracture surgeries included in the study, the operation note for the surgery was written by a medical officer in 37 cases (42.05%) and by an orthopaedic trainee in 29 cases (32.95%). A specialist wrote the operation note in 21 cases (23.86%), while a consultant documented the note in only one case (1.14%) (Table 1). This distribution highlights the varying levels of experience among the individuals responsible for documenting these surgeries, which may have implications for the quality and detail of the operative notes.

**Table 1:** Overview of personnel documenting operative notes for femoral neck fractures

Personnel documenting the operative notes	Number (n)	Percentage (%)
Consultant	1	1.14
Specialist	21	23.86
Resident trainee	29	32.95
Medical officer	37	42.05

The study additionally found that a number of important parameters were highly frequently recorded, making them the elements in the operative notes that were most consistently recorded. In particular, more than 90% of the notes included information about the surgeon and assistant's names, the procedure name, the operative diagnosis, the operative procedure, prosthesis details, deep vein thrombosis (DVT) prophylaxis, antibiotic prophylaxis, and signatures (Table 2).

**Table 2:** An overview of the documentation frequency of the parameters DVT: deep vein thrombosis

Parameter assessed	Documentation frequency (n)	Documentation frequency (%)
Identification	66	75
Time of procedure	13	14.7
Elective/emergency procedure	0	0
Name of surgeon and assistant	85	96.6
Name of theatre anaesthetist	0	0
Name of procedure	88	100
Type of incision	22	25
Operative diagnosis	88	100
Operative findings	41	46.6
Complications encountered	5	8.7
Any extra procedure performed with reason	0	0
Details of closure technique	56	63.6
Anticipated blood loss	17	19.3
Prosthesis details	80	90.9
Antibiotic prophylaxis	81	92
DVT prophylaxis	82	93.2
Detailed post op instructions	80	90.9
Signature	100	100

Other parameters, however, were notably lacking in every form that was analyzed. None of the operative notes included the name of the theatre anaesthetist, information about whether the procedure was emergency-caused or elective, or details about any further procedures that were carried out and their justifications (Table 2). Subsequent investigation showed that a number of other parameters were the least frequently recorded aspects, with documentation occurring in less than 50% of the operative notes. These parameters included the procedure's duration, the type of incision used, the operative findings, the expected blood loss, details of the closure technique, and the existence or absence of surgical complications (Table 2).

## Discussion

The ability to take precise and comprehensive operative notes is a critical component of quality assurance in surgery and is one of the most critical skills that a surgeon must possess [5]. This is because the proper documentation of a surgical procedure is essential for the postoperative care of the patient [5]. The absence of an appropriately completed operation note in this study is indicative of the suboptimal quality of patient perioperative detail documentation, necessitating enhancement.

The results highlight the variation in documentation practices and underscore the need for targeted interventions to enhance the accuracy and completeness of operative notes. Consistently recording all pertinent information can greatly enhance surgical team communication, promote better postoperative care, and ultimately result in better patient outcomes. 

The comprehensive analysis of 88 operative notes, according to the RCS guidelines, has provided significant insights into the necessity of improving operative note documentation practices. The findings revealed that certain parameters, such as surgeon and assistant names, operative diagnosis, operative procedure, prosthesis details, DVT and antibiotic prophylaxis, and signatures, were documented in over 90% of the notes. This high frequency of recording suggests a strong adherence to documentation protocols and standards among the surgical team. It indicates that these critical details are consistently captured, which can enhance communication, ensure continuity of care, and improve patient outcomes by providing comprehensive and reliable medical records.

Additionally, parameters such as the time of the procedure, type of incision, operative findings, anticipated blood loss, closure technique specifics, and whether complications were encountered were documented in less than 50% of the operative notes. These aspects, although less frequently recorded, are crucial for providing a comprehensive account of the surgical procedure and for informing postoperative care and future surgical interventions. Conversely, the absence of the theatre anaesthetist’s name, details regarding elective/emergency settings, and any additional procedures performed with reasons in all notes highlights a significant gap in the documentation process.

These omissions in surgical procedure notes could have serious implications. They may hinder continuity of care and decision-making for follow-up treatments, as critical details such as surgical findings and complications are not documented. Additionally, incomplete records may pose legal risks, as accurate documentation is crucial for defending against malpractice claims or litigation. Moreover, poor documentation can lead to misunderstandings or miscommunication among healthcare providers, potentially compromising patient safety and outcomes. It is essential to ensure thorough and accurate documentation practices to uphold patient care standards, legal compliance, and effective communication in healthcare settings.

Particularly in the context of surgical documentation, the process of altering established habits presents distinctive obstacles. Surgeons frequently miss certain details in operation notes, which are instead recorded in alternative forms by key members of the surgical team, such as the operating room nurse or anaesthetist [6]. Antibiotic prophylaxis and the duration of the surgery are typically recorded by anaesthetists on intraoperative charts [7]. Nevertheless, it is strongly advised to directly integrate these details into the operation notes to enhance clarity and accessibility. This practice guarantees that all parties involved in patient care have access to comprehensive information, which facilitates more informed decision-making processes and promotes smoother communication.

Research consistently demonstrates that computer-based templates or typed notes are preferred over handwritten ones [8]. Our centre (GCOST) is a distinguished tertiary hospital that serves the local population and the surrounding villages. It has not yet implemented electronic operation notes due to financial constraints. There is a strong conviction that electronic note documentation establishes higher standards for the completion of operative procedures [9].

Audits conducted in Nigeria and other African regions have shown similar, and sometimes worse, outcomes [10-12]. Conversely, audits from developed countries have exhibited slightly better results [13,14]. In addition, Sweed et al. [15] conducted an audit in Kuwait that revealed that 20% of the operative notes they examined were incomplete, contained illegible parts, and contained confusing abbreviations. A study from Sudan found that the names of the anaesthetists were rarely documented (13.9%) [11]; similarly, our study found that the anaesthetists’ names were mentioned in none of the notes.

The study's limitations include focusing on a single hospital, which may restrict the generalizability of the findings to other contexts. Furthermore, the limited sample size may not offer a thorough examination of documentation practices and their variations. The scope is further restricted by the exclusive emphasis on fracture neck of femur procedures, which fails to consider the diverse array of surgical procedures conducted in a typical orthopaedic setting.

## Conclusions

The study shows notable shortcomings in the quality of operating notes, emphasizing the need for systematic educational programs focused on improving the documentation abilities of medical officers and orthopaedic trainees. These professionals have the main responsibility of documenting operative information. Therefore, tailored training is crucial to enhance documentation standards and guarantee thorough and accurate medical records. Operative note types could be standardized by implementing structured templates that adhere to RCS standards. These templates would enhance documentation by establishing a clear framework, ensuring that all necessary information is included, and maintaining uniformity. This would address the existing inconsistencies in documentation practices.

Regular audits and feedback sessions are crucial for identifying recurring issues in operative notes and implementing effective corrections. Workshops and seminars should be scheduled regularly to train medical officers and trainees regarding efficient documentation methods, emphasizing the importance of comprehensive records in orthopaedic surgery.
